# Prediction of intraocular pressure reduction after phacoemulsification surgery: A prospective observational study

**DOI:** 10.1097/MD.0000000000047730

**Published:** 2026-02-20

**Authors:** Sinem Karabulut, Müjdat Karabulut

**Affiliations:** a Ophthalmology Department, Mugla Training and Research Hospital, Mugla, Turkey.

**Keywords:** anterior chamber depth, intraocular pressure, lens thickness, lens vault, phacoemulsification

## Abstract

We aimed to determine the predictive value of the anterior segment and lens structures for intraocular pressure (IOP) reduction after uncomplicated phacoemulsification surgery. This prospective observational study included 133 nonglaucomatous open-angle eyes with uncomplicated cataracts. The preoperative anterior segment and lens structure measurements were performed. The predictive values of preoperative IOP, anterior chamber depth (ACD), axial length, lens thickness (LT), lens vault (LV), anterior chamber volume, iridocorneal angle, anterior chamber angle 500/750, and angle opening distance 500/750 for IOP reduction were analyzed. IOP reduction was significantly correlated with LV, ACD, LT, and preoperative IOP (*R* = 0.292, *P* = .003; *r* = −0.218, *P* = .030; *R* = 0.205, *P* = .043; and *R* = 0.660, *P* < .001, respectively). LV (*F* [1, 97] = 9.051, *P* = .003), ACD (*F* [1, 97] = 4.841, *P* = .030), LT (*F* [1, 97] = 3.829, *P* = .043), and preoperative IOP (*F* [1, 97] = 74.791, *P* < .001) were significant predictors of IOP reduction. Preoperative IOP, LV, LT, and ACD were significant predictors of IOP reduction in nonglaucomatous open-angle eyes after uncomplicated phacoemulsification surgery.

## 1. Introduction

Intraocular pressure (IOP) reduction after phacoemulsification surgery is well-known and substantial, although the exact mechanism and extent of the reduction are still poorly understood. Most studies have been designed to predict the change in IOP after phacoemulsification surgery and to demonstrate the relationship between IOP change and the anterior segment and lens structures. Changes in some anatomical and biometric structures, such as anterior chamber depth (ACD), anterior chamber angle (ACA), angle opening distance (AOD), and anterior chamber volume, were related to IOP differences after phacoemulsification surgery.^[1,2]^ It has been reported that the IOP-lowering effect of phacoemulsification surgery was significantly affected by baseline IOP and antiglaucomatous medication use in eyes with glaucoma.^[3,4]^

Various reports have been published regarding the relationship between IOP change after surgery and the anterior segment and crystalline lens structures. Some studies have shown that IOP reduction is primarily associated with anterior chamber structures, such as AOD 750 and ACD.^[5]^ On the other hand, others have reported that lens parameters such as lens thickness (LT), lens vault (LV), and anterior vault might be used to predict IOP reduction.^[2]^ However, most studies have been conducted on glaucomatous eyes. The limited number of studies conducted on normal eyes inspired us to study these eyes.

This prospective study aimed to determine the predictive value of both the anterior segment and lens structures in IOP reduction after uncomplicated phacoemulsification surgery in nonglaucomatous open-angle eyes.

## 2. Materials and methods

This prospective, observational study included 133 eyes of 133 patients with cataract-related complaints such as blurred vision, glare, light sensitivity, and monocular diplopia. After a complete ophthalmologic examination, including gonioscopic examination, which was performed by the same observer (SK) in the 150 to 200 lux photopic condition with Posner 4-mirror gonioscopic lens, open-angle eyes (grades 3 and 4 in all 4 quadrants for the Shaffer system) with cataract-related visual acuity (VA) reduction (VA < 0.3 decimal) were obtained for the study. Patients whose VA reduction was related to other ocular pathologies, such as refractive, corneal, vitreal, retinal, or optic nerve pathologies, and amblyopia, were excluded. Eyes with narrow angles in at least 1 quadrant on gonioscopic examination (grade 2 or less for the Shaffer system), iris and iridocorneal anomalies, peripheral anterior synechiae, complicated (inflammatory, pseudoexfoliative, congenital, traumatic etiology and phacodonesis, zonular weakness, or dialysis findings), dense cataracts that caused optical biometric measurements to fail, history of previous intraocular surgery, any glaucomatous findings in their visual field and retinal nerve fiber layer analysis, and IOP >30 mm Hg were not included. A standard, uncomplicated phacoemulsification surgery (2.4-mm clear corneal incisions, 4- to 6-mm manual continuous curvilinear capsulorhexis, hydrodissection, phacoemulsification, hydrophobic acrylic foldable single-piece intraocular lens [IOL] [AcrySof SA60AT, Alcon] implantation into the capsular bag, removal of the viscoelastic) was performed by the same surgeon (SK) using the Centurion Vision System (Alcon Laboratories, Inc., Irvine). Nepafenac 0.3% and a moxifloxacin/dexamethasone combination of eye drops were prescribed postoperatively once and 6 times a day for 1 week and tapered for ~3 weeks.

Eyes with intraoperative (anterior or posterior capsule rupture, vitreous loss, iridodialysis, sulcus IOL implantation, etc) or postoperative complications (IOL dislocation, corneal decompensation, capsular phimosis, anterior-posterior synechia, glaucoma, etc) and elevated IOP compared with baseline were also excluded.

The same observer (SK) measured the patients’ preoperative and postoperative 3rd-month IOP using a Goldmann applanation tonometer at the same time period to minimize diurnal fluctuations. The average of 3 consecutive measurements was recorded.

All images were recorded preoperatively 1 day before the surgery by the same expert in the same room with the 150 to 200 lux photopic condition and nondilated pupil. ACD, axial length, LT, anterior chamber volume, and iridocorneal angle were automatically measured using an IOLMaster 700 optical biometer (Carl Zeiss Meditec Ltd., Jena, Germany) and a Pentacam Scheimpflug camera (OCULUS Optikgeräte GmbH, Wetzlar, Germany). Spectralis (Heidelberg Engineering, Inc., Heidelberg, Germany) anterior segment optical coherence tomography was used to image iridocorneal angles. The measurement was accepted as reliable if the standard deviation was <27 and <38 µm for axial length and LT, respectively, for the IOLMaster optical biometer, a Q score of >15 for Spectralis, and an image quality score of >95% for the Pentacam.

In the Spectralis images, a glaucoma expert (SK) manually measured AOD 500 and 750 as perpendicular distances from the corneal point (500 and 750 μm away from the scleral spur) to the iris and recorded them as temporal/nasal AOD 500 and AOD 750. Temporal/nasal ACA 500 and 750 were measured manually using software embedded in the device, as the angle between a point on the posterior corneal surface that was 500 and 750 μm away from the scleral spur and iris surface.^[6]^ The vertical distance between the lens apex and the horizontal line binding the nasal and temporal scleral spurs was recorded as the LV.

A previous power analysis using GPower 3.1.9.7 software (Heinrich-Heine-Universität Düsseldorf, Düsseldorf, Germany) showed that 133 sample sizes provided 0.999 power (linear multiple regression statistic test, α error probability level of .05).

The results were recorded as means ± standard deviations and counts/percentages for continuous and categorical variables. Pearson correlation coefficient and multiple regression analysis were used to assess correlations and predictive values. Because we had a single dependent variable and multiple independent variables, we used multiple regression analysis. Statistical significance was set at *P* < .05.

The study was approved by the Muğla Sitki Koçman University Medical and Health Sciences Ethics Committee (protocol/decision number: 230106/110). All participants provided informed consent, and the principles of the Declaration of Helsinki were followed throughout the study.

## 3. Result

This study included 133 eyes from 133 patients who met the inclusion criteria. Thirty-one patients were excluded due to intraoperative and postoperative complications (such as anterior and posterior capsular rupture, sulcus IOL placement, vitreous loss, and increased postoperative IOP). Four patients did not complete their last visit.

Images of 98 (74%) eyes of 98 patients (45 [46%] female, 53 [54%] male) remained for the study. Post hoc power analysis revealed that 98 sample sizes provided 0.994 power at an α error probability level of .05.

The mean age was 71.05 ± 7.43 (48–88 years). The mean preoperative and postoperative IOP was 16.32 ± 4.11 (10–26) mm Hg and 12.43 ± 3.10 (5–21) mm Hg, respectively. The mean IOP reduction was 3.88 ± 2.87 (1–14) mm Hg. Preoperative anterior segment measurements and lens structures are presented in Table 1.

**Table 1:** Preoperative parameters and IOP measurement of the eyes (n = 98).

	Min.	Max.	Mean ± SD
Preop IOP	10	26	16.32 ± 4.11
Postop IOP	5	21	12.43 ± 3.10
IOP reduction	1	14	3.88 ± 2.87
LV	50	1338	400.14 ± 229.59
ACD	2.14	3.98	3.05 ± 0.38
LT	3.62	5.40	4.64 ± 0.35
AOD 500			
Nasal	184	633	374.45 ± 97.27
Temporal	133	695	361.95 ± 111.91
AOD 750			
Nasal	247	831	480.22 ± 123.94
Temporal	235	821	458.61 ± 136.20
ACA 500			
Nasal	35	45	39.93 ± 4.82
Temporal	34	42	38.93 ± 4.85
ACA 750			
Nasal	36	46	40.70 ± 5.71
Temporal	35	45	41.13 ± 3.82
ICA	36.70	46.60	40.71 ± 5.90
AL	21.28	25.37	23.35 ± 0.79
ACV	52	216	125.53 ± 35.13

ACA = anterior chamber angle, ACD = anterior chamber depth, ACV = anterior chamber volume, AL = axial length, AOD = angle opening distance, ICA = iridocorneal angle, IOP = intraocular pressure, LT = lens thickness, LV = lens vault, Max. = maximum, Min. = minimum, n = number, Postop = postoperative, Preop IOP = preoperative IOP, SD = standard deviation.

**Figure 1.**
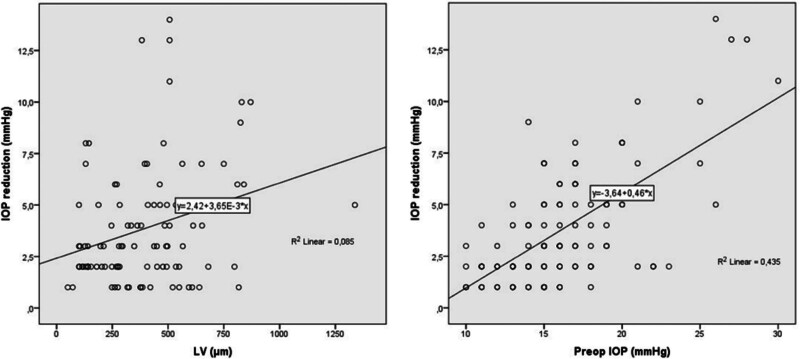
The regression scatterplot of IOP reduction with Preop IOP and LV. IOP = intraocular pressure, LV = lens vault, Preop IOP = preoperative IOP.

**Figure 2.**
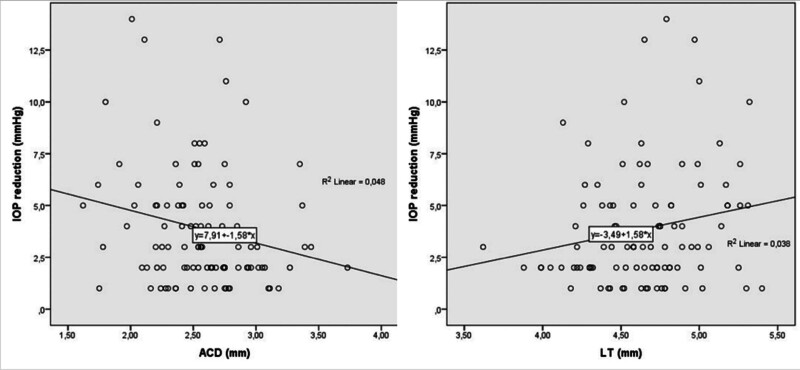
The regression scatterplot of IOP reduction with ACD and LT. ACD = anterior chamber depth, IOP = intraocular pressure, LT = lens thickness.

IOP reduction was significantly correlated with LV, ACD, LT, and preoperative IOP (*R* = 0.292, *P* = .003; *r* = −0.218, *P* = .030; *R* = 0.205, *P* = .043; *R* = 0.660, *P* < .001, respectively) (Table 2). Regression analysis showed that LV (*F* [1, 97] = 9.051, *P* = .003), ACD (*F* [1, 97] = 4.841, *P* = .030), LT (*F* [1, 97] = 3.829, *P* = .043), and preoperative IOP (*F* [1, 97] = 74.791, *P* < .001) were significant predictors of IOP reduction (Tables 3 and 4). The regression equation demonstrated that for each 1-mm increase in LV and LT, and 1-mm Hg increase in preoperative IOP, the predicted IOP reduction was ~2.4, 1.9, and 3.2 mm Hg, respectively. Additionally, for each 1-mm reduction in ACD, the predicted IOP reduction was ~6.3 mm Hg. Figures 1 and 2 illustrate the relationship between the dependent variable (*y*-axis: IOP reduction) and the independent variables (*x*-axis: LV, preoperative IOP, ACD, LT).

**Table 2:** Correlation analysis of IOP reduction and preoperative ocular parameters (n = 98).

	IOP reduction	
	Pearson correlation	Sig.
Preop IOP	0.660	**<.001**
LV	0.292	**.003**
ACD	−0.218	**.030**
LT	0.205	**.043**
AOD 500		
Nasal	−0.043	0.673
Temporal	−0.072	0.481
AOD 750		
Nasal	−0.037	0.719
Temporal	−0.031	0.762
ACA 500		
Nasal	0.031	0.764
Temporal	0.003	0.980
ACA 750		
Nasal	0.018	0.857
Temporal	0.030	0.766
ICA	0.049	0.628
AL	−0.048	0.634
ACV	−0.013	0.898

The Pearson correlation coefficient was used to assess correlations. The significant *P*-values are shown in bold.

ACA = anterior chamber angle, ACD = anterior chamber depth, ACV = anterior chamber volume, AL = axial length, AOD = angle opening distance, ICA = iridocorneal angle, IOP = intraocular pressure, LT = lens thickness, LV = lens vault, n = number, Preop IOP = preoperative IOP, sig. = significance.

**Table 3:** Regression analysis results (n = 98).

Predictors	*R*	*R* ^2^	Adj. *R*^2^	df	*F*	Sig.
				Reg./Res.		
Preop IOP	0.660	0.435	0.430		74.791	**<.001**
LV	0.292	0.085	0.076	1/97	9.051	**.003**
ACD	−0.218	0.048	0.038		4.841	**.030**
LT	0.205	0.038	0.033		3.829	**.043**
AOD 500						
Nasal	−0,043	0.002	0.008		0.179	0.673
Temporal	−0,072	0.005	0.005		0.500	0.481
AOD 750						
Nasal	−0,037	0.001	0.009		0.130	0.719
Temporal	−0,031	0.001	0.009		0.092	0.762
ACA 500						
Nasal	0.031	0.001	0.009		0.091	0.764
Temporal	0,003	0.000	0.010		0.001	0.980
ACA 750						
Nasal	0,018	0.000	0.010		0.033	0.857
Temporal	0,030	0.001	0.009		0.089	0.766
ICA	0,049	0.002	0.008		0.237	0.628
AL	−0,048	0.002	0.008		0.228	0.634
ACV	−0,013	0.000	0.010		0.017	0.898

Dependent variable: IOP reduction. Multiple regression analysis showed that preop IOP, LV, ACD, and LT were significant predictors of IOP reduction.

ACA = anterior chamber angle, ACD = anterior chamber depth, ACV = anterior chamber volume, Adj. = adjusted, AL = axial length, AOD = angle opening distance, ICA = iridocorneal angle, IOP = intraocular pressure, LT = lens thickness, LV = lens vault, n = number, Preop IOP = preoperative IOP, Sig. = significance.

**Table 4:** Regression analysis results (n = 98).

Predictors	Unstandardized coefficients		Standardized coefficients	95% Confidence interval for B		*t*
	*B*	Std. Err.	Beta	Lower bound	Upper bound	
Preop IOP (constant)	−3.643	0.897		−5.423	−1.864	−4.063
	0.461	0.053	0.660	0.355	0.567	8.648
LV (constant)	2.416	0.560		1.305	3.527	4.317
	0.004	0.001	0.292	0.001	0.006	3.008
ACD (constant)	7.914	1.856		4.231	11.598	4.265
	−1.575	0.716	−0.218	−2.996	−0.154	−2.200
LT (constant)	−3.489	3.336		−10.984	4.006	−0.924
	1.585	0.810	0.195	−0.023	3.192	1.957
AOD 500						
Nasal	−0.001	0.003	−0.043	−0.007	0.005	−0.424
Temporal	−0.002	0.003	−0.072	−0.007	0.003	−0.707
AOD 750						
Nasal	−0.001	0.002	−0.037	−0.006	0.004	−0.361
Temporal	−0.001	0.002	−0.031	−0.005	0.004	−0.303
ACA 500						
Nasal	0.015	0.050	0.031	−0.084	0.114	0.302
Temporal	0.001	0.050	0.003	−0.098	0.100	0.025
ACA 750						
Nasal	0.009	0.051	0.018	0.092	0.110	0.181
Temporal	0.015	0.050	0.030	−0.084	0.114	0.298
ICA	0.024	0.049	0.049	−0.074	0.122	0.487
AL	−0.176	0.368	−0.048	−0.906	0.554	−0.478
ACV	−0.001	0.008	−0.013	−0.018	0.015	−0.129

Dependent variable: IOP reduction. The regression equations of the significant predictors with constants and other parameters.

ACA = anterior chamber angle, ACD = anterior chamber depth, ACV = anterior chamber volume, AL = axial length, AOD = angle opening distance, ICA = iridocorneal angle, IOP = intraocular pressure, LT = lens thickness, LV = lens vault, n = number, Preop IOP = preoperative IOP, sig. = significance.

## 4. Discussion

Estimating the extent to which IOP decreases after phacoemulsification surgery and the factors affecting this decrease is essential. The primary aim of this surgery extends beyond improving visual symptoms. Preventing cataract-related complications, maintaining eye health, regulating IOP, protecting patients from drug-related side effects, and reducing drug-related economic losses are key concerns. Therefore, predicting the extent of IOP reduction after cataract surgery and the extent to which the patient will benefit from this surgery provides opinions on surgical planning.

A prospective study with nonglaucomatous and open-angle eyes showed a mean decrease in IOP of 2.03 ± 2.42 mm Hg after uncomplicated phacoemulsification surgery.^[7]^ Other studies reported this IOP reduction as 2.19 and 2.04 mm Hg after uncomplicated phacoemulsification surgery.^[8,9]^ One of the predicting factors related to IOP reduction after phacoemulsification surgery is preoperative IOP. Wang et al^[3]^ reported in a sizeable sample-sized study that preoperative IOP was a decisive predictive factor for IOP reduction after phacoemulsification surgery. They found an IOP reduction of more than 6 mm Hg in eyes with preoperative IOP ≥ 21 mm Hg. This IOP reduction is almost equivalent to that of minimally invasive glaucoma surgery. On the other hand, postoperative IOP reduction was much lower in eyes with a preoperative IOP range of 12 to 14 mm Hg. This study found a mean of ~3.88 ± 2.87 mm Hg reduction in eyes with a mean preoperative IOP of ~16 mm Hg. We also found that every 1-mm Hg increase in preoperative IOP resulted in a 3.2-mm Hg postoperative IOP reduction.

Nongpiur et al ^[10]^ defined the LV as the vertical distance between the apex of the lens and the horizontal line connecting the nasal and temporal scleral spurs. They thought that it could be used as a predictor of IOP reduction after cataract surgery. In a prospective study, Huang et al^[11]^ reported a significant association between LV and IOP reduction after phacoemulsification. Furthermore, in a recent study, Bahadur et al^[12]^ reported that LV is highly associated with IOP reduction after phacoemulsification surgery. Like previous studies, a significant relationship was found between LV and IOP reduction in the current study. We also found that every 1-mm increase in LV resulted in a 2.4-mm Hg IOP reduction.

LT and ACD are other predictive factors that affect IOP changes after cataract surgery. Hsu et al^[7]^ found that lens position, computed from LT and ACD, was an accessible predictor of IOP change after phacoemulsification surgery. Additionally, some studies have confirmed that ACD and LT are inversely and positively related to IOP changes.^[13,14]^ We also found an inverse and positive relation between ACD, LT, and IOP change.

Most studies have shown improved angle parameters, which resulted in IOP reduction after phacoemulsification surgery.^[15,16]^ However, it is still controversial that these parameters might predict IOP reduction after phacoemulsification surgery. Various results have been published showing the relationship between ACA-related parameters, such as ACA 500 to 750, AOD 500 to 750, and IOP changes after phacoemulsification surgery, depending on the preoperative angle status. Sarkar et al^[17]^ reported that AOD could significantly predict the IOP decrease after phacoemulsification surgery. On the other hand, Sengupta et al^[18]^ reported that AOD was unrelated to IOP reduction after phacoemulsification surgery in open-angle eyes. Similarly, Zhou et al^[19]^ showed that the preoperative ACA parameters did not predict IOP reduction after phacoemulsification surgery. We also did not find a significant correlation between IOP reduction and ACA-related parameters (ACA 500–750, AOD 500–750) in eyes with an open angle after uncomplicated phacoemulsification surgery.

Diurnal IOP fluctuation refers to the changes in IOP that occur throughout the day. Some studies have shown that IOP increases in the morning or late afternoon and decreases in the early afternoon.^[20,21]^ Additionally, IOP reduction after cataract surgery usually stabilizes within 2 to 4 months.^[22]^ In this study, IOP measurements were taken at the same time period of the day to minimize variations due to diurnal fluctuations and at the postoperative 3rd month to allow IOP reduction to stabilize. In the current study, we enrolled only open-angle and nonglaucomatous eyes to define the pure effects of uncomplicated phacoemulsification and IOL implantation surgery on IOP reduction and to determine the predictive value of lens and anterior segment structures on IOP reduction. This study has some limitations. Thirty-one patients were excluded due to intraoperative and postoperative complications (such as anterior and posterior capsular rupture, sulcus IOL placement, vitreous loss, and increased postoperative IOP), and 4 patients did not complete their final visit. This percentage may seem high, suggesting a potential bias. However, this study aimed to determine the factors affecting IOP changes in nonglaucomatous open-angle eyes with uncomplicated cataracts, without intraoperative or postoperative complications. Furthermore, patients who did not attend follow-up visits were also excluded due to incomplete data. Therefore, patients were excluded only if they met the exclusion criteria, thereby preventing any potential bias. The lack of correlation between IOP reduction and ACA-related parameters may be due to variability in manually measured angle parameters, as well as the exclusion of eyes with narrow angles. Another limitation of this current study was that it was conducted at a single center, which may limit its external validity. Additionally, we did not consider the correlation (multicollinearity) among independent variables. Long-term follow-up with a large sample size and evaluation of glaucomatous, complicated cataracts, or closed-angled eyes might have affected the results.

In conclusion, preoperative IOP, LV, LT, and ACD were predictors of IOP reduction; however, angle parameters were not associated with IOP reduction in nonglaucomatous open-angle eyes with uncomplicated cataracts. Future multicenter and multisurgeon studies might enhance external validity.

## Author contributions

**Conceptualization:** Sinem Karabulut, Müjdat Karabulut.

**Data curation:** Sinem Karabulut.

**Formal analysis:** Sinem Karabulut.

**Investigation:** Sinem Karabulut.

**Methodology:** Sinem Karabulut, Müjdat Karabulut.

**Software:** Sinem Karabulut.

**Supervision:** Sinem Karabulut, Müjdat Karabulut.

**Validation:** Sinem Karabulut.

**Visualization:** Sinem Karabulut.

**Writing – original draft:** Sinem Karabulut.
